# RALP1 is essential for schizont maturation and erythrocyte invasion in *Plasmodium falciparum*

**DOI:** 10.1186/s13071-026-07329-w

**Published:** 2026-03-06

**Authors:** Jing Wu, Zuping Zhang, Jiayao Pang, Wenyu Yang, Chandara Ngim, Peiyi Li, Jingru Ye, Bin Tian, Xinyu Cheng, Fei Wang, Qingfeng Zhang, Xiaomin Shang

**Affiliations:** 1 Department of Parasitology, Xiangya School of Basic Medicine, Central South University, Changsha, 410013 China; 2 Laboratory of Molecular Parasitology, State Key Laboratory of Cardiology and Research Center for Translational Medicine, Shanghai East Hospital; Key Laboratory of Pathogen-Host Interaction (Tongji University), Ministry of Education; Clinical Center for Brain and Spinal Cord Research, School of Medicine, Tongji University, Shanghai, 200120 China; 3 Hunan Provincial Key Laboratory of Immunology and Transmission Control on Schistosomiasis (The Third People’s Hospital of Hunan Province), Yueyang, 414000 China; 4 Clinical Laboratory Department, Changsha Municipal Center for Disease Control and Prevention, Changsha, 410004 China

**Keywords:** *Plasmodium falciparum*, Merozoite, Erythrocyte invasion, Rhoptry protein

## Abstract

**Background:**

*Plasmodium falciparum* merozoite invasion of erythrocytes is an essential step in the asexual blood-stage cycle and a major target for antimalarial intervention. Rhoptry neck proteins play key roles in the formation and function of the tight junction, yet many remain poorly characterized. RALP1, a conserved rhoptry neck-associated leucine zipper-like protein, has been proposed to participate in erythrocyte binding and invasion. Conventional gene disruption attempts have been unsuccessful, suggesting that RALP1 may be essential for parasite survival. Nevertheless, its precise role and broader molecular impact during intraerythrocytic development remain to be fully elucidated.

**Methods:**

We generated a 3 × HA-tagged conditional knockdown line (*ralp1-ha-glmS*) using CRISPR-Cas9-mediated homologous recombination. RALP1 abundance and subcellular localization were evaluated by Western blotting and immunofluorescence assays. Effects on parasite growth, schizont maturation, merozoite invasion, and merozoite numbers were assessed using tightly synchronized cultures and established invasion and cytological assays. Transcriptomic changes following GlcN-induced RALP1 knockdown were analyzed by RNA-seq at early ring and schizont stages. Sequence-based structural and epitope features were examined using IUPred2A, ANCHOR2, AlphaFold3, NetMHCpan, and NetMHCIIpan.

**Results:**

Precise integration of the *ha-glmS* cassette enabled GlcN-inducible reduction of RALP1 protein levels, most prominently in schizonts. RALP1 knockdown reduced parasite proliferation, impaired schizont maturation, decreased merozoite numbers, and lowered erythrocyte invasion efficiency. RNA-seq showed limited effects in early rings but widespread downregulation of invasion- and host-parasite interaction-related genes in schizonts after correction for glucosamine-responsive transcripts, with GO enrichment highlighting processes related to host cell interaction, biological adhesion, and membrane-associated components. Sequence-based analyses indicated that RALP1 contains extensive intrinsically disordered regions with multiple predicted interaction motifs, while predicted B- and T-cell epitope hotspots concentrated within the C-terminal RBC-binding domain. AlphaFold3 modeling yielded low global confidence (pTM = 0.23), consistent with a primarily disordered architecture.

**Conclusions:**

RALP1 is required for normal schizont maturation and efficient erythrocyte invasion in *P. falciparum*. Its partial knockdown perturbs transcription of key invasion ligands and apical components, indicating a broader role in preparing merozoites for host-cell entry. The extensive disorder, epitope-rich C-terminal region, and essential function of RALP1 highlight its potential as a candidate for therapeutic or vaccine targeting.

**Graphical Abstract:**

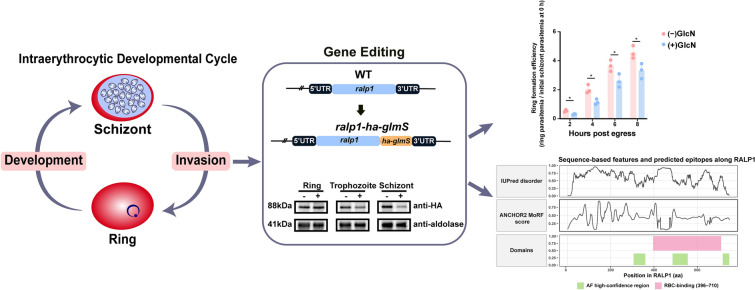

**Supplementary Information:**

The online version contains supplementary material available at 10.1186/s13071-026-07329-w.

## Background

Malaria continues to pose a major global public health challenge, with the World Health Organization (WHO) estimating approximately 282 million cases and about 610,000 deaths worldwide in 2024 [1]. The disease burden falls disproportionately on sub-Saharan Africa, where children under 5 account for most fatalities [2]. Among the five *Plasmodium* species that infect humans, *Plasmodium falciparum* is the most virulent. It undergoes a 48-h intraerythrocytic cycle that progresses from early rings to trophozoites and subsequently to multinucleated schizonts. Upon host cell rupture, each schizont releases 16–32 merozoites, which further propagate infection by invading uninfected erythrocytes. Malaria pathogenesis is thus driven by the repetitive process of merozoite invasion of erythrocytes and the relentless asexual replication cycle. The invasion process represents a well-characterized target for both vaccine development and monoclonal antibody therapies [3, 4].

The *Plasmodium* invasion cascade begins with low-affinity, reversible merozoite attachment to the erythrocyte surface [5, 6]. After apical reorientation, invasion proceeds by forming an electron-dense tight junction (TJ), through which the parasite drives internalization via an actin-myosin motor system [7–9]. Concurrently, the merozoite becomes enveloped by a parasitophorous vacuole (PV), providing a protected intracellular niche [6, 10]. Successful invasion requires sequential secretion from three apical secretory organelles—micronemes, rhoptries, and dense granules—with microneme discharge occurring first, followed by rhoptry release and dense-granule secretion after PV formation [11].

Rhoptries are the largest and most specialized secretory organelles of the apical complex. Their biogenesis initiates approximately 40-h post-invasion through sequential fusion of Golgi-derived vesicles, generating paired apically positioned structures [12]. Rhoptries exhibit a bipartite architecture: the neck region contains early-acting invasion factors such as the RON complex, which mediates TJ formation, whereas the bulb compartment stores later-acting effectors, including RAP proteins that participate in PV establishment [13, 14]. Rhoptry-secreted proteins thus play central roles in host-cell entry and subsequent intracellular development, making them attractive candidates for blood-stage vaccine design.

RALP1 (rhoptry-associated leucine zipper-like protein 1, PF3D7_0722200) is a conserved rhoptry neck protein that contains a signal peptide, a Gly/Glu-rich region, and a leucine-zipper-like domain [15]. It is specifically expressed in schizonts and merozoites and exhibits a C-terminal erythrocyte-binding region, implicating this domain in host-parasite interactions during invasion [15]. RALP1 translocates from the rhoptry neck to the moving junction during invasion, further suggesting a direct role in TJ formation and entry [16]. As a conserved invasion-associated protein, RALP1 has been proposed as a promising blood-stage vaccine antigen [16, 17]. Importantly, *ralp1* has been reported to be refractory to genetic disruption, suggesting a potential essential role during intraerythrocytic development and necessitating conditional genetic approaches for functional investigation [18, 19].

In this study, we employed a ribozyme-guided conditional knockdown strategy, combined with invasion phenotyping, RNA-seq transcriptional profiling, structural prediction, and epitope analysis, to elucidate the functional role of RALP1 during the asexual blood-stage cycle of *P. falciparum*.

## Methods

### Parasite culture

*Plasmodium falciparum* 3D7 parasites were maintained in fresh O⁺ human erythrocytes (< 2 weeks old) stored at 4 °C, at 2% hematocrit, using complete RPMI 1640 medium (Gibco) supplemented with 0.5% Albumax I (Invitrogen), 0.2% sodium bicarbonate, 25 mM HEPES, and 50 μg/ml hypoxanthine. Cultures were incubated at 37 °C under standard low-oxygen conditions (5% O_2_, 5% CO_2_, 90% N_2_). Ring-stage parasites were routinely synchronized using 5% D-sorbitol. For experiments requiring tighter synchronization, including transcriptomic or invasion assays, late schizonts were enriched using a 40%/70% Percoll-sorbitol gradient, followed by a second 5% sorbitol treatment 5 h after reinvasion to obtain a highly synchronized culture within a ~ 5-h developmental window[20].

### Plasmid construction

The pL6CS plasmid was modified according to previously described CRISPR/Cas9 methods by introducing homology sequences targeting the *ralp1* locus [21]. The specific sgRNA sequence targeting *ralp1* was designed using the CRISPR guide design tool available at PlasmoDB (https://plasmodb.org/plasmo/app). Synthesized oligonucleotides (Sangon Biotech) were diluted to 100 μM, annealed by gradient cooling to form double-stranded DNA, and ligated into the pL6CS backbone between the XhoI and PstI sites using seamless cloning enzyme (Vazyme, C112). To investigate the function of RALP1, approximately 500-bp fragments flanking the stop codon of *ralp1* were amplified by PCR. An *ha-glmS* cassette was inserted between these two fragments through overlap-extension PCR, and silent mutations were introduced into the sgRNA target site to prevent re-cutting by Cas9. The modified homology block was then inserted into the pL6CS vector between the SgsI and BspT I sites using seamless cloning. The recombinant plasmids were transformed into *Escherichia coli* XL10 for amplification, verified by Sanger sequencing. Plasmids were purified using a plasmid maxi-prep kit (TransGen, EM123). The sequences of the sgRNA and primers used for plasmid construction are provided in Additional file 1: Table S1.

#### Generation of transgenic lines

For transfection, 150 μl of uninfected fresh erythrocytes was mixed with 150 μl containing 100 μg of purified pL6CS-*ralp1-ha-glmS* plasmid and 100 μg of pUF1-Cas9 plasmid, dissolved together in 150 μl of ultrapure water. Electroporation was performed using a Bio-Rad electroporator under standard conditions, followed by the immediate addition of Percoll-enriched schizont-stage parasites. Cultures were maintained with daily medium changes[22]. When parasitemia reached approximately 10% (after ~ 2 replication cycles), WR99210 (2.5 nM) and DSM1 (2 μg/ml; Invitrogen) were added, and drug-containing medium was replaced daily. Once no viable parasites were observed in Giemsa-stained thin blood smears, medium changes were reduced to twice per week. Viable parasites typically reappeared approximately 3 weeks after transfection. After expansion, genomic DNA was extracted using a commercial kit (TIANGEN, DP304), and the regions flanking the designed integration sites were amplified by PCR using *P. falciparum* 3D7 genomic DNA as a wild-type control. PCR products were verified by DNA sequencing to confirm correct genomic integration. Diagnostic PCR did not detect the presence of wild-type alleles in the edited parasite population. Based on this result, parasite cloning was not performed, and all subsequent experiments were carried out using the bulk (non-clonal) transgenic population. Primer sequences are listed in Additional file 1: Table S1.

#### Growth curve analysis

The *ralp1-ha-glmS* strain was tightly synchronized to a 5-h developmental window. Synchronized ring-stage parasites were seeded at 0.1% parasitemia in six-well plates at 2% hematocrit, with or without 5 mM glucosamine (GlcN). Parasites were cultured for four consecutive replication cycles, and Giemsa-stained thin blood smears were prepared during each cycle to monitor parasitemia. Data from three independent biological replicates were plotted and analyzed using GraphPad Prism 9.

#### Western blotting

Parasites were tightly synchronized to a 5-h developmental window at the ring stage and then cultured at 1% parasitemia and 2% hematocrit, with or without 5 mM glucosamine (GlcN). In the second replication cycle, 200 μl of ring-, trophozoite-, and schizont-stage samples were collected. Samples were resuspended in PBS, and infected erythrocytes were lysed with 0.15% saponin to release parasites. Pellets were collected by centrifugation, resuspended in an appropriate volume of PBS, and mixed with an equal volume of 2 × Laemmli sample buffer (Beyotime, P0015). Samples were heated at 100 °C for 10 min in a metal heater and centrifuged, and the supernatants were collected as total protein extracts. Protein lysates were separated on 10% SDS-polyacrylamide gels and transferred onto Immobilon-P PVDF membranes (Sangon Biotech, F619534). Proteins were transferred at 220 mA for 1 h at 4 °C. Membranes containing total protein were blocked with 5% non-fat milk in PBST (0.1% Tween-20 in PBS) for 2 h at room temperature, followed by overnight incubation at 4 °C with primary antibodies diluted in PBST:rabbit anti-HA (1:5000; HUABIO, HA721750) and rabbit anti-aldolase (1:3000; Abcam, ab207494). After three washes with PBST, membranes were incubated for 1 h at room temperature with HRP-conjugated secondary antibodies—goat anti-rabbit IgG (1:8000; HUABIO, HA1001). Following three additional washes, signals were developed using ECL substrate (PUMOKE, PMK0448) and visualized with the ChemiDoc™ XRS + imaging system (Bio-Rad). All Western blot experiments were performed at Central South University.

#### Immunofluorescence assays

Immunofluorescence assays were performed as previously described[23] to determine the subcellular localization of RALP1. Parasites were tightly synchronized and collected at the schizont stage. After lysis of infected erythrocytes with 0.15% saponin, parasites were immediately fixed on ice in ice-cold 4% paraformaldehyde for 30 min. Fixed parasites were adhered to glass slides, permeabilized with 0.1% Triton X-100 in PBS for 5 min, and blocked with 1% BSA for 1 h at room temperature. Samples were incubated with rabbit anti-HA primary antibody (1:500; HUABIO, HA721750) for 1 h at room temperature, followed by incubation with iFluor™ 488-conjugated goat anti-rabbit IgG secondary antibody (1:500; HUABIO, HA1121) for 1 h. Slides were washed three times with PBS between each incubation. Nuclei were stained with DAPI. Fluorescence imaging was performed on a STELLARIS 5 confocal microscope (Leica Microsystems) using a 100 × oil immersion objective. Images were processed in ImageJ.

#### RNA-seq library preparation and sequencing

*Ralp1-ha-glmS* parasite cultures were tightly synchronized to a 5-h developmental window using Percoll gradient centrifugation followed by sorbitol treatment. Parasites were cultured with or without 5 mM glucosamine (GlcN) and harvested in the next replication cycle at the ring stage (10–12 hpi) and schizont stage (45–48 hpi). Parasite pellets were homogenized in Transzol reagent (TransGen, ER501), vortexed thoroughly, and stored at −80 °C until further processing. Total RNA was extracted using the TransZol Up Plus RNA Kit (TransGen, ER501) according to the manufacturer’s instructions. For strand-specific RNA-seq library preparation, 500 ng of high-quality RNA from each sample was used with the VAHTS Universal V10 RNA-seq Library Prep Kit (Vazyme, NR616). Poly(A) + mRNA was enriched using oligo(dT) magnetic beads, followed by fragmentation to ~ 300 to 400 nt, first- and second-strand cDNA synthesis, adapter ligation, and 14 cycles of PCR amplification. Final libraries were assessed for quality using a Qubit 2.0 fluorometer (Invitrogen) and an Agilent 2100 Bioanalyzer (Agilent Technologies). Paired-end sequencing (150 bp) was performed on an Illumina NovaSeq 6000 platform by BMK Biotechnology (Beijing, China).

#### RNA-seq data processing and analysis

Raw reads were quality-checked and trimmed using TrimGalore (v0.6.6) to remove adapters and low-quality bases. Cleaned reads were aligned to the *Plasmodium falciparum* reference genome (PlasmoDB v64) using HISAT2 (v2.2.1). SAM files were converted and sorted into BAM format using Samtools (v1.12), and duplicates were marked and removed with Picard Tools (v2.26.0). Gene-level read counts were derived using featureCounts (v2.0.1). Differential expression analysis was then performed with the DESeq2 package (v1.46.0) in R (v4.4.2), with two biological replicates per condition. Genes with baseMean < 1 were excluded from the analysis. Differentially expressed genes (DEGs) were defined as those with | log_2_ (fold change) |> 1 and *P* < 0.05. Gene Ontology (GO) enrichment was performed using annotated data from PlasmoDB.

#### Invasion versus egress assay

The invasion versus egress assay was performed based on previously published methods [24]. Parasites maintained under normal culture conditions without GlcN treatment were synchronized, and the synchronized ring-stage parasites were divided into two groups with or without 5 mM GlcN. Parasites were then allowed to develop into mature schizonts, which were isolated using Percoll gradient centrifugation. The enriched schizonts were incubated at 37 °C for 4 h in pre-warmed RPMI medium supplemented with the PKG inhibitor ML10 (Sigma, Z544396), which was dissolved in DMSO to a 50 μM stock concentration, to block parasite egress and maintain tight synchronization at the late schizont stage [25]. After incubation, parasites were washed twice with pre-warmed RPMI medium, the supernatant was removed, and 2 μl of the enriched schizonts was immediately added to 80 μl of pre-warmed red blood cells at 2% hematocrit. Thin blood smears were prepared every 2 h over an 8-h period (0, 2, 4, 6, and 8 h) and stained with Giemsa solution, and parasitemia was calculated by counting at least 5000 erythrocytes per smear. The assay was performed in three independent biological replicates, and the data were analyzed using GraphPad Prism 9.

#### Sequence-based structural feature prediction and epitope analysis

Structural and immunogenic features of RALP1 were analyzed using multiple sequence-based computational tools. Intrinsically disordered regions (IDRs) were predicted with IUPred2A, and molecular recognition features (MoRFs) were identified using ANCHOR2. Linear B-cell epitope scores were obtained using BepiPred through the Immune Epitope Database (IEDB; https://www.iedb.org/). T-cell epitope propensity was first evaluated using general IEDB tools and subsequently refined using NetMHCpan-4.1 (MHC-I) and NetMHCIIpan-4.0 (MHC-II). Results from disorder prediction, MoRF identification, epitope scoring, AlphaFold3 high-confidence regions, and the known RBC-binding region (aa 396–710) were integrated for downstream visualization and interpretation. All the analyses were performed in R (v4.4.2) using custom scripts.

#### MHC-I and MHC-II epitope prediction across HLA alleles

To comprehensively assess the T-cell epitope landscape of RALP1, potential peptide binders to human MHC molecules were predicted using tools implemented on the IEDB platform. For MHC class I, all possible 9-mer peptides were scanned using NetMHCpan-4.1, and binding affinities were categorized into strong (≤ 0.5%), weak (0.5–2%), and non-binders (> 2%). Distribution plots and positional mappings were generated accordingly. For MHC class II, peptide binding to representative HLA-DRB1 alleles was predicted using NetMHCIIpan-4.0, analyzing 15-mer peptides classified into strong (≤ 2%), weak (2–10%), and non-binding (> 10%) categories. Epitope intervals predicted across multiple HLA alleles were compared to assess sequence regions with broad HLA coverage. All computational analyses were conducted in R (v4.4.2) using custom visualization pipelines.

## Results

### *Ralp1* is conserved and targeted by invasion-related ApiAP2 factors

**Fig. 1:**
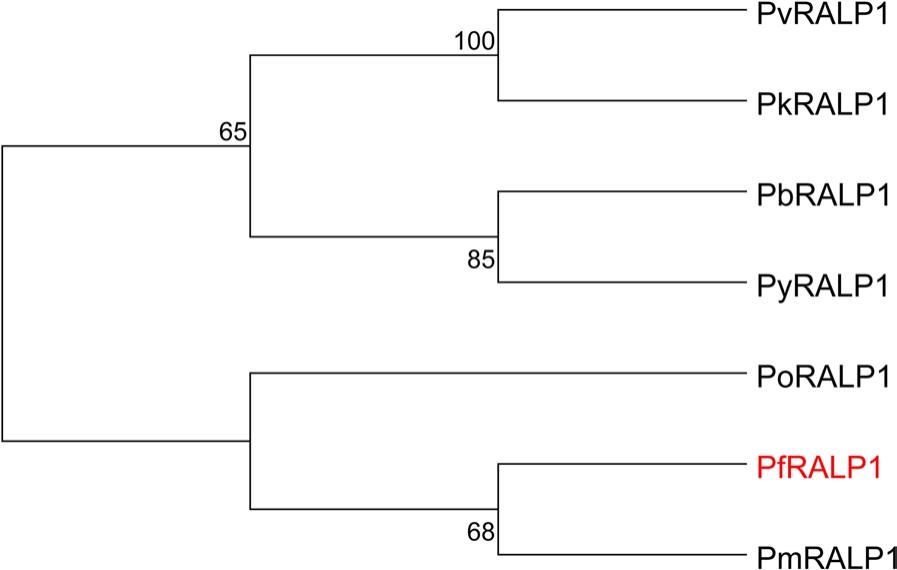
Evolutionary relationship of RALP1 across *Plasmodium* species. Maximum-likelihood phylogenetic tree constructed using full-length RALP1 amino acid sequences from representative *Plasmodium* species, including *P. vivax (Pv), P. knowlesi (Pk), P. berghei (Pb), P. yoelii (Py), P. ovale (Po), P. falciparum (Pf)*, and *P. malariae (Pm)*. Node values indicate bootstrap support (%) based on 1000 replicates.

The open reading frame (ORF) of *ralp1* comprises 2250 base pairs and encodes a ~ 87.9 kDa protein containing a leucine zipper-like domain. To evaluate the sequence conservation of *ralp1* among different *Plasmodium* species, we performed amino acid sequence alignments of *ralp1* orthologs from several representative parasites. Phylogenetic analysis revealed that *ralp1* is highly conserved across both human- and rodent-infecting *Plasmodium* species, particularly among major human malaria parasites such as *P. falciparum* and *P. malariae*, supporting the hypothesis that RALP1 may serve as a potential cross-species vaccine target (Fig. 1). RALP1 expression displays stage specificity, peaking during the early ring and late schizont stages, which reflects its tight transcriptional regulation [26]. As the largest family of transcription factors in *Plasmodium*, ApiAP2 transcription factors are known to critically regulate key biological processes including gametocytogenesis and host cell invasion [27]. Since RALP1 acts as an effector protein during merozoite invasion of erythrocytes, we examined published datasets to assess the binding of ApiAP2 transcription factors—previously implicated in invasion-related gene regulation—at the *ralp1* genomic locus [28, 29]. This analysis identified strong binding by AP2-O5 and AP2-EXP, weaker binding by AP2-I, and absence of promoter binding for several other transcription factors. Collectively, these data support a model in which *ralp1* expression is coordinately regulated by AP2-O5, AP2-EXP, and AP2-I, underscoring its importance in the erythrocyte invasion process (Additional file 4: Fig. S1).

### Conditional knockdown of RALP1 reduces schizont-stage protein levels and impairs asexual blood-stage growth

**Fig. 2:**
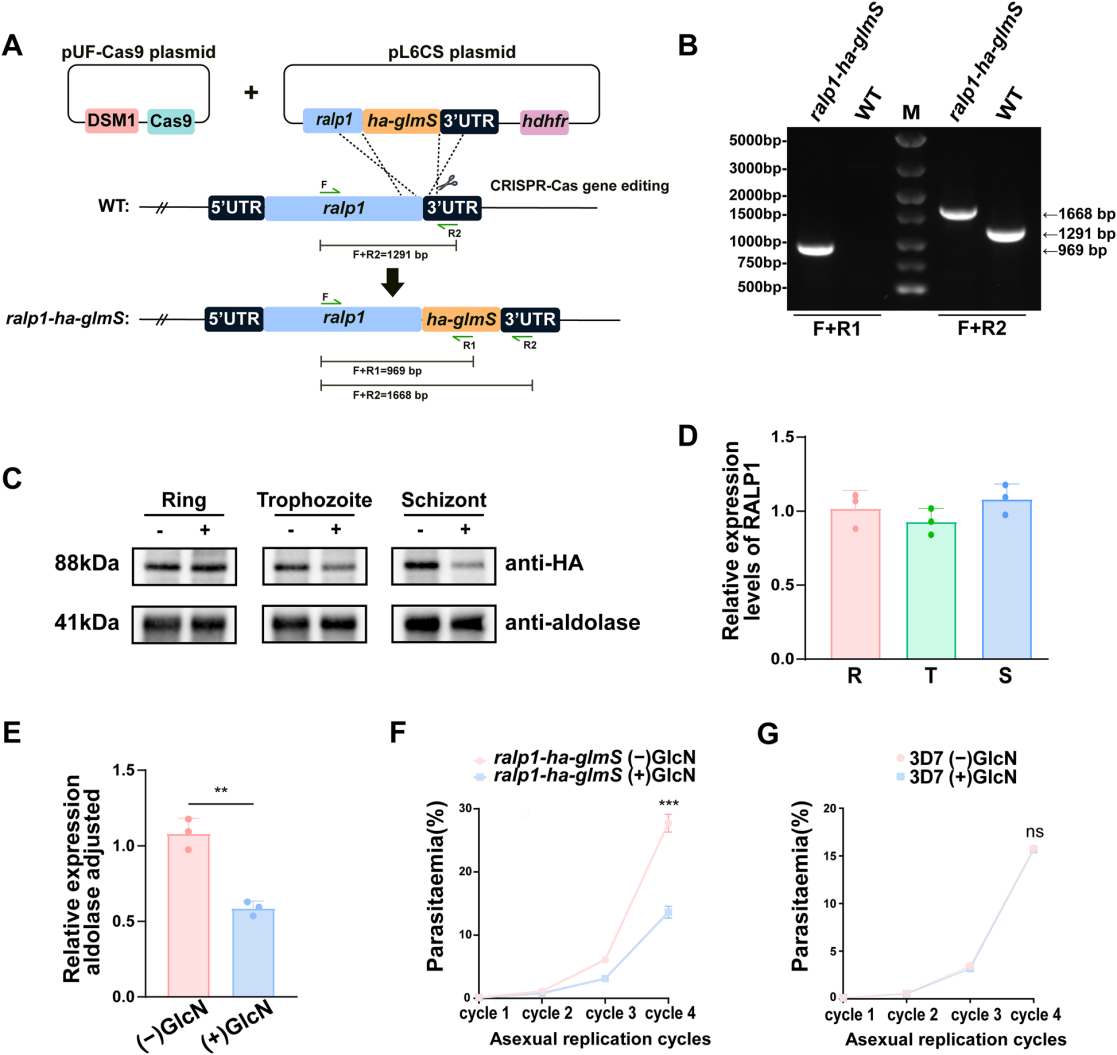
Generation and validation of the *ralp1-ha-glmS* conditional knockdown parasite line.** A** Schematic of the CRISPR-Cas9 strategy used to generate the *ralp1-ha-glmS* parasite. A pUF-Cas9 plasmid expressing Cas9 under the control of the DSM1 selection marker and a donor pL6CS plasmid containing the C-terminal *ha-glmS* tag and *hdhfr* cassette were co-transfected. Homologous recombination replaced the endogenous 3′ region of *ralp1* with the *ha-glmS* module. Primer pairs (F + R1 and F + R2) used for genotyping are indicated. **B** Diagnostic PCR confirming successful integration at the *ralp1* locus. The *ralp1-ha-glmS* parasites show the expected 969-bp (F + R1) and 1668-bp (F + R2) bands, whereas wild-type (WT) parasites show the endogenous 1291-bp band. **C** Western blot analysis detecting 3 × HA-tagged RALP1 expression across the ring (R), trophozoite (T), and schizont (S) stages in the presence (+) or absence (−) of GlcN. Aldolase serves as the loading control. **D** Relative RALP1 protein levels across R/T/S stages under − GlcN conditions, quantified from Western blot analyses. This panel is intended to illustrate the stage-dependent expression pattern of RALP1 and does not represent a statistical comparison between stages. **E** Quantification of RALP1 protein levels in *ralp1-ha-glmS* parasites with (+ GlcN) or without (− GlcN) glucosamine treatment, normalized to aldolase. Glucosamine treatment results in an approximately ~ 40% reduction in RALP1 protein abundance (*P* < 0.01, Student’s t-test). **F** Growth analysis of *ralp1-ha-glmS* parasites with (+ GlcN) or without (− GlcN) glucosamine over four asexual replication cycles. RALP1 knockdown leads to a reduced proliferation rate compared with untreated parasites. **G** Growth analysis of wild-type 3D7 parasites cultured with (+ GlcN) or without (− GlcN) glucosamine, showing no significant difference in parasite growth. Data are presented as mean ± SD from three independent experiments. Statistical significance is indicated as follows: *P* < 0.05; *P* < 0.01; *P* < 0.001; ns, not significant.

To investigate the function of RALP1, we generated a conditional knockdown parasite line by inserting a 3 × HA-tagged *glmS* ribozyme into the 3′ end of the endogenous *ralp1* locus via CRISPR-Cas9-mediated homologous recombination (Fig. 2A). Diagnostic PCR using primer pairs spanning the 3′ integration junctions (F + R1 and F + R2) yielded amplicons of the expected sizes exclusively in the transgenic line, confirming precise and complete integration of the *ha-glmS* cassette at the *ralp1* locus (Fig. 2B). Western blot analysis with an anti-HA antibody showed that RALP1 is expressed throughout the intraerythrocytic cycle. Under (−) GlcN conditions, analysis of synchronized parasites revealed a stage-dependent expression pattern, with RALP1 protein levels being relatively higher at the schizont stage than in the ring and trophozoite stages (Fig. 2C, D). Upon glucosamine (GlcN) treatment, activation of the *glmS* ribozyme led to a clear reduction in RALP1 protein abundance. Quantitative densitometric analysis focused on schizont-stage parasites—the stage at which RALP1 expression is highest—showed an approximately 40% decrease in RALP1 protein levels compared with untreated controls (Fig. 2C, E). These data indicate that the *ralp1-ha-glmS* parasite line is correctly engineered, expresses 3 × HA-tagged RALP1, and allows effective GlcN-inducible knockdown of RALP1 in schizonts. To assess the impact of partial RALP1 knockdown on parasite growth, tightly synchronized cultures of the *ralp1-ha-glmS* parasites were maintained with or without GlcN and monitored over four consecutive asexual replication cycles. Parasites cultured in the presence of GlcN showed consistently lower parasitemia than untreated controls, with a marked divergence from the third cycle onward (Fig. 2F). In contrast, parallel growth analysis of the parental 3D7 strain under identical (−) GlcN and (+) GlcN conditions revealed no significant difference in parasitemia across the same replication cycles (Fig. 2G). Together, these results indicate that RALP1 plays an important role in intraerythrocytic parasite growth.

### RALP1 knockdown impairs schizont maturation, erythrocyte invasion, and merozoite egress

**Fig. 3:**
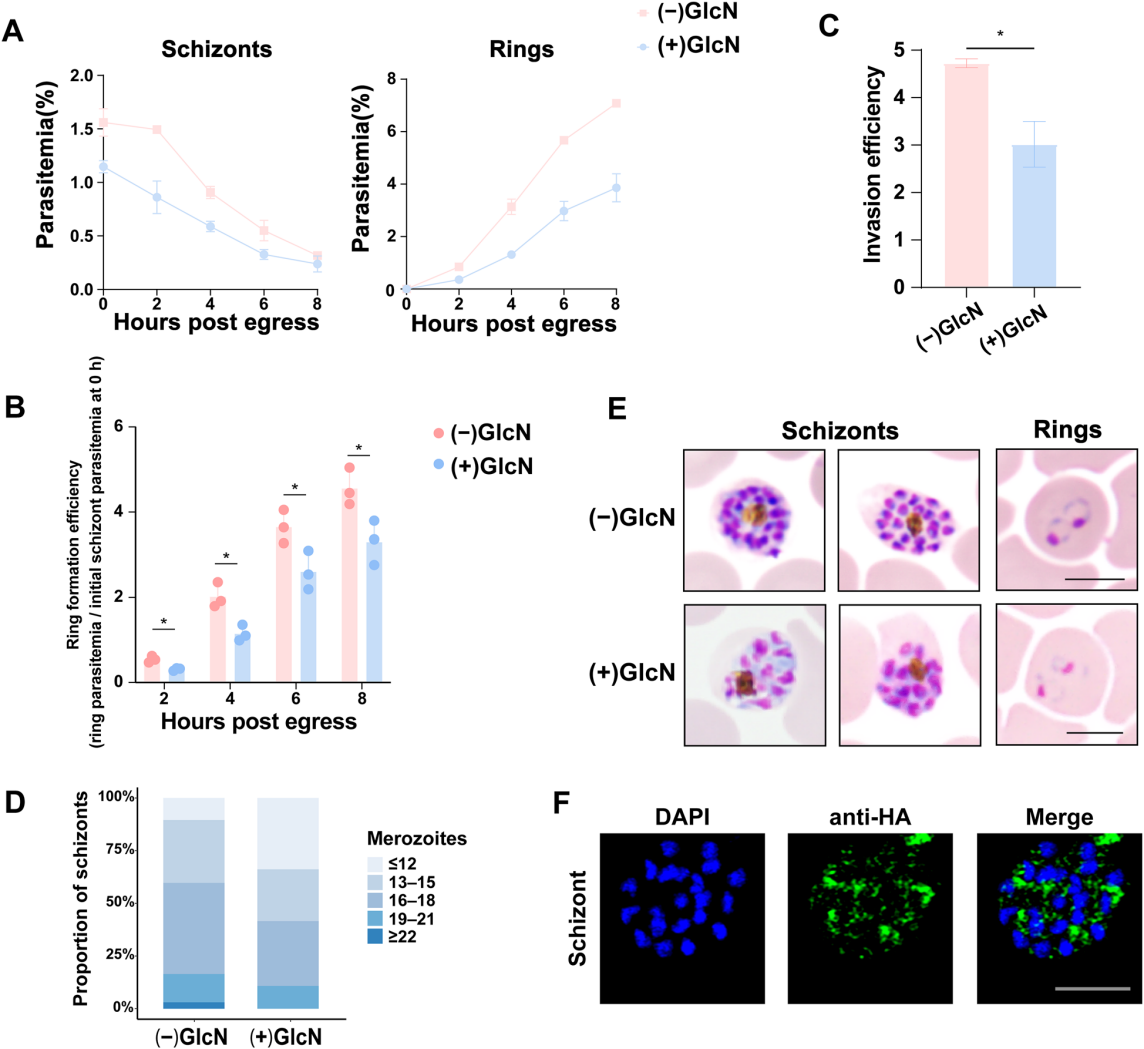
Partial reduction of RALP1 impairs merozoite egress, invasion, and schizont maturation. **A** Time course of schizont decline (left) and ring appearance (right) following egress in *ralp1-ha-glmS* parasites cultured with (+ GlcN) or without GlcN (− GlcN). Parasitemia was monitored every 2 h over an 8-h period. **B** Quantification of ring formation efficiency following egress. Ring-stage parasitemia was normalized to the initial schizont parasitemia at 0 h post-egress, thereby controlling for differences in starting schizont levels between − GlcN and + GlcN conditions. **C** Invasion efficiency of *ralp1-ha-glmS* parasites treated with (+ GlcN) or without (− GlcN) glucosamine, measured by newly formed rings after synchronized schizont rupture. **D** Distribution of merozoite numbers per mature schizont in − GlcN and + GlcN parasites, presented as stacked bar charts. Bars indicate the proportions of schizonts containing ≤ 12, 13–15, 16–18, 19–21, or ≥ 22 merozoites. **E** Giemsa-stained images of representative schizonts and rings in − GlcN and + GlcN groups. Scale bars: 5 μm. **F** Immunofluorescence images of schizonts probed with anti-HA antibody (green) and DAPI (blue), showing 3 × HA-tagged RALP1 expression and localization. Scale bar: 5 μm. Statistical significance is indicated as follows: *P < 0.05; **P < 0.01; ***P < 0.001.

To determine whether RALP1 contributes to erythrocyte invasion, tightly synchronized late schizonts were inhibited with ML10, a PKG inhibitor, to block parasite egress and arrest parasites at the late schizont stage; parasites were then allowed to egress and and reinvade in the presence or absence of GlcN. RALP1 knockdown parasites (+ GlcN) exhibited reduced post-egress parasitemia, in both the remaining schizont population and the newly formed rings, across the 8-h observation period (Fig. 3A). To control for differences in initial schizont parasitemia between conditions, ring formation efficiency was quantified by normalizing ring-stage parasitemia at each time point to the initial schizont parasitemia measured at 0 h post-egress. Using this normalized metric, RALP1 knockdown parasites (+ GlcN) consistently showed reduced ring formation efficiency compared with untreated controls (Fig. 3B), indicating impaired conversion of released merozoites into ring-stage parasites. To specifically assess merozoite invasion independent of schizont number and egress efficiency, invasion efficiency was directly measured based on the proportion of newly formed rings following synchronized schizont rupture. Consistent with the ring formation analysis, parasites cultured in the presence of GlcN showed significantly reduced invasion efficiency compared with untreated controls (Fig. 3C). To further assess schizont maturation, we quantified merozoite numbers per mature schizont. RALP1 knockdown parasites displayed a shift toward schizonts containing fewer merozoites, whereas control parasites more frequently exhibited schizonts with higher merozoite counts (Fig. 3D), suggesting impaired schizont maturation. Giemsa-stained thin smears revealed morphological abnormalities in schizonts under RALP1 knockdown (+ GlcN) conditions, including less compact segmentation and reduced merozoite density, which were not observed in untreated controls (Fig. 3E). Immunofluorescence assays showed that 3 × HA-tagged RALP1 localized around the perinuclear region of schizonts (Fig. 3F), consistent with its involvement in late-stage parasite development. Together, these findings demonstrate that partial knockdown of RALP1 impairs schizont maturation, reduces merozoite numbers, and diminishes erythrocyte invasion efficiency. These invasion-related defects prompted further analysis of RALP1-dependent transcriptional changes.

### RALP1 knockdown predominantly disrupts schizont-stage transcription and suppresses invasion-associated genes

**Fig. 4:**
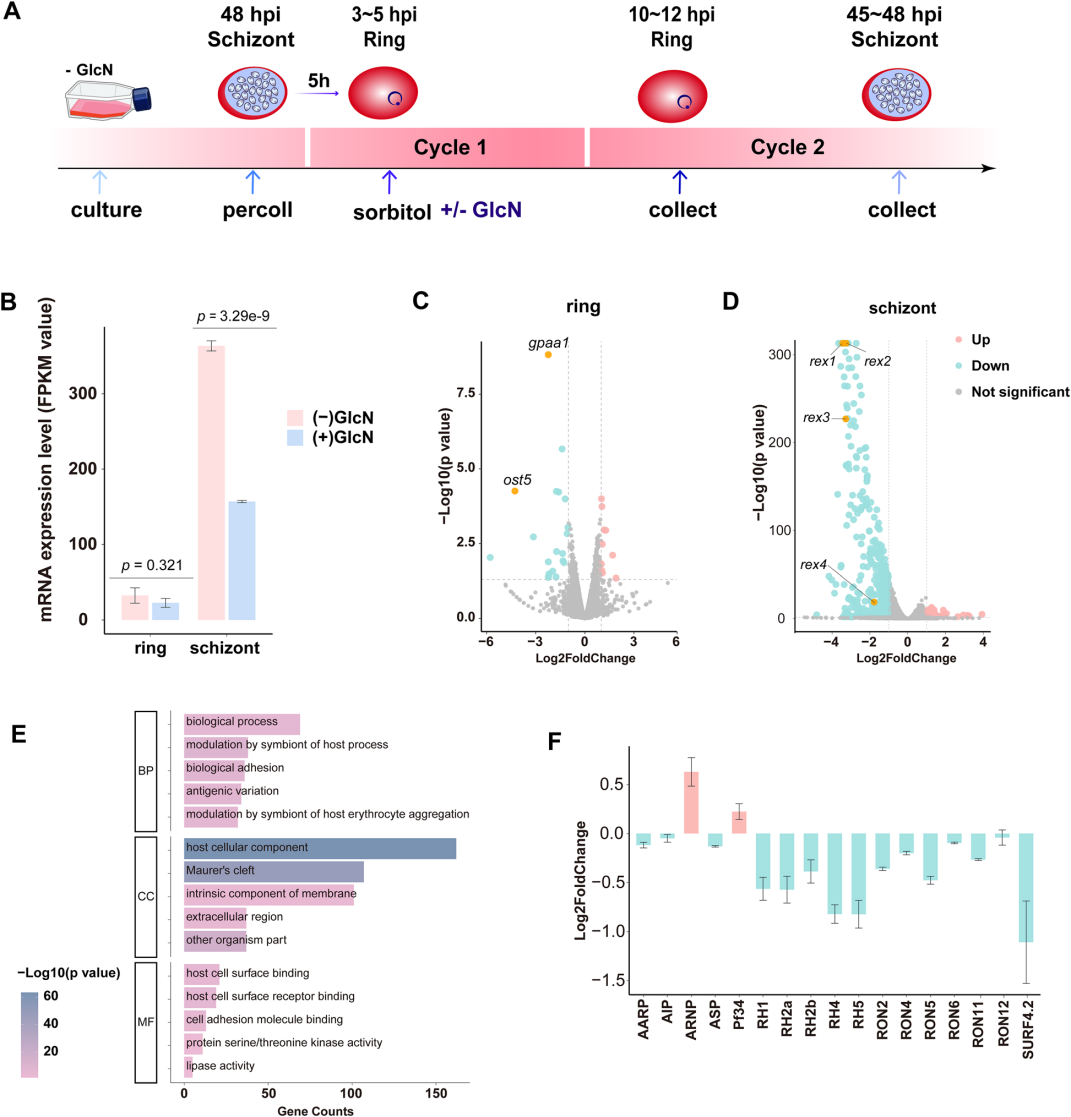
Transcriptomic analysis of *ralp1-ha-glmS* parasites following glucosamine-induced knockdown. **A** Schematic overview of the RNA-seq sampling workflow. Parasites were cultured to 48 hpi schizonts (cycle 1) and purified by Percoll, then synchronized with sorbitol and incubated with or without glucosamine (± GlcN). In cycle 2, early ring-stage parasites (10–12 hpi) and schizonts (45–48 hpi) were collected for comparative transcriptomic analysis. **B** mRNA expression levels of *ralp1* (FPKM) in ring and schizont stages under − GlcN and + GlcN conditions. Significant reduction of *ralp1* transcripts was detected in schizonts after GlcN treatment, while ring-stage levels remained unchanged. **C** Volcano plot showing differential gene expression in ring-stage parasites between − GlcN and + GlcN groups after correction for GlcN-responsive genes. Selected significantly altered genes are highlighted. **D** Volcano plot showing differential gene expression in schizont-stage parasites between − GlcN and + GlcN groups after correction for GlcN-responsive genes. **E** GO enrichment analysis of differentially expressed genes in schizonts. Representative enriched terms are shown for biological process (BP), cellular component (CC), and molecular function (MF). **F** Log2 fold-change values of key invasion-related genes, including EBA, RH, and RON family members, comparing − GlcN versus + GlcN conditions.

To characterize transcriptional changes associated with RALP1 knockdown, synchronized *ralp1-ha-glmS* parasites were harvested at the early ring (10–12 hpi) and schizont (45–48 hpi) stages from cultures grown with or without GlcN (Fig. 4A). At the time of sample collection, parasite developmental stages were confirmed by Giemsa-stained thin blood smears, showing comparable stage distributions between − GlcN and + GlcN conditions (Additional file 5: Fig. S2). To minimize confounding effects of glucosamine treatment, genes identified as GlcN-responsive in the parental 3D7 strain were excluded from subsequent analyses, and all downstream results are based on this GlcN-corrected gene set (Additional file 6: Fig. S3 A; Additional file 2: Table S2) [30]. Analysis of *ralp1* transcript levels revealed no significant difference between − GlcN and + GlcN conditions at the ring stage, whereas a marked reduction was observed at the schizont stage following GlcN treatment (Fig. 4B), consistent with the stage-dependent knockdown efficiency of the *glmS* system. After GlcN correction, differential expression analysis revealed limited transcriptional changes at the ring stage, with 8 genes upregulated and 17 genes downregulated under + GlcN conditions (Fig. 4C). In contrast, schizont-stage parasites exhibited extensive transcriptional changes upon RALP1 knockdown, with 55 genes upregulated and 360 genes downregulated (Fig. 4D; Additional file 6: Fig. S3B; Additional file 3: Table S3). Gene Ontology enrichment analysis of downregulated schizont genes showed significant enrichment in biological processes associated with host-parasite interactions, including modulation of host processes, biological adhesion, and antigenic variation. Enriched cellular component terms included host cellular components, Maurer’s clefts, intrinsic components of membranes, and extracellular regions. Molecular function analysis highlighted enrichment in host cell surface binding, host cell surface receptor binding, cell adhesion molecule binding, and protein serine/threonine kinase activity (Fig. 4E). Focusing on rhoptry neck proteins [14], partial knockdown of RALP1 resulted in decreased expression of numerous invasion ligands and microneme proteins, including Pf41, RH1, RH2b, RH4, RON2, RON5, RON6, and SURFIN4.2 (Fig. 4F). Only a few genes, such as AARP, showed increased expression. Together, these data indicate that partial knockdown of RALP1 leads to widespread transcriptional alterations in schizonts, particularly affecting genes involved in host cell interaction and erythrocyte invasion.

### RALP1 contains extensive intrinsically disordered regions and concentrated epitope-rich segments across its RBC-binding domain

**Fig. 5:**
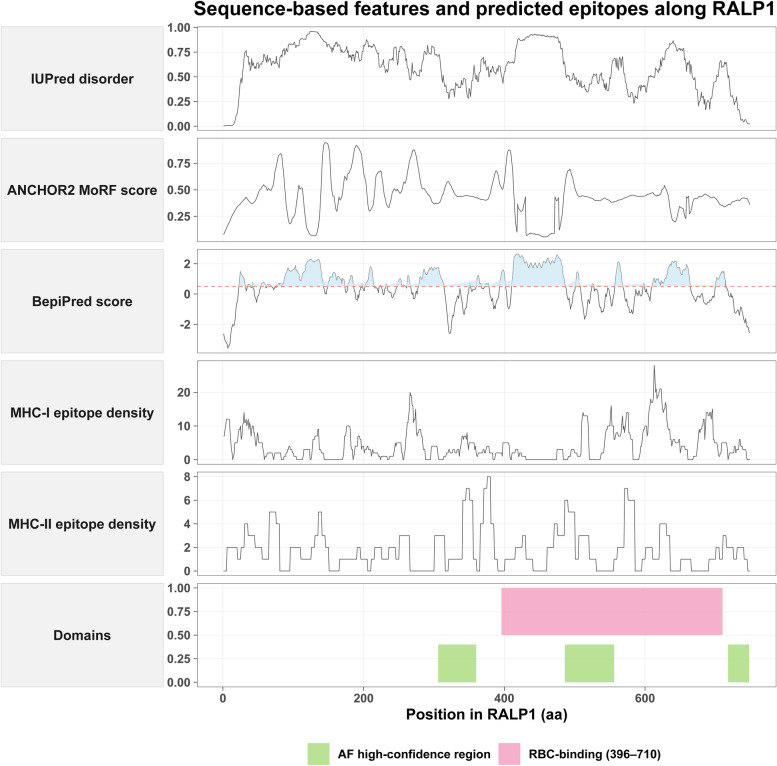
Sequence-based structural features and predicted epitope landscape of RALP1. This composite plot summarizes multiple computational predictions across the full-length RALP1 amino acid sequence, including IUPred-disordered regions, ANCHOR2-predicted molecular recognition features (MoRFs), BepiPred B-cell epitope propensity, and predicted densities of MHC class I and class II epitopes. The lower panel shows domain annotations, including AlphaFold high-confidence regions (green) and the reported red blood cell–binding region (residues 396–710, pink). Together, these analyses provide an overview of intrinsic disorder, potential binding motifs, and antigenic hotspots along RALP1.

To characterize the sequence features of *ralp1* and assess its potential immunogenic regions, we systematically analyzed intrinsic disorder, predicted molecular recognition features (MoRFs), B-cell epitopes, and T-cell epitope densities along the full-length protein (Fig. 5). IUPred disorder profiling revealed that RALP1 contains large intrinsically disordered regions (IDRs), particularly within the N-terminal and central regions, whereas the C-terminal RBC-binding domain (aa 396–710) exhibited comparatively reduced disorder. Consistently, ANCHOR2 analysis identified multiple MoRF peaks embedded within these disordered segments. To further evaluate whether these sequence-derived features correspond to structural properties, we predicted the 3D structure of RALP1 using AlphaFold3 (Additional file 7: Fig. S4). The resulting model displayed an overall low predicted template modeling score (pTM = 0.23), indicating limited confidence in the global fold and suggesting that a substantial portion of the protein may lack a stable tertiary structure, consistent with the presence of extensive IDRs. Notably, several short regions corresponding to AlphaFold high-confidence segments overlapped with the C-terminal RBC-binding domain. BepiPred predicted several continuous B-cell epitope-rich segments predominantly within moderately disordered central regions of the protein. Mapping class I and class II T-cell epitope densities (NetMHCpan and NetMHCIIpan) further revealed epitope hotspots concentrated within the C-terminal RBC-binding domain, coinciding with AlphaFold-predicted structured subregions. Collectively, these analyses highlight RALP1 as a hybrid protein composed of widespread disordered regions interspersed with localized structured elements, with its RBC-binding domain exhibiting the highest density of B- and T-cell epitopes. These features suggest potential immunogenicity and functional relevance of this region in host-parasite interactions.

### RALP1 contains multiple conserved T-cell epitope hotspots enriched within the C-terminal RBC-binding region

**Fig. 6:**
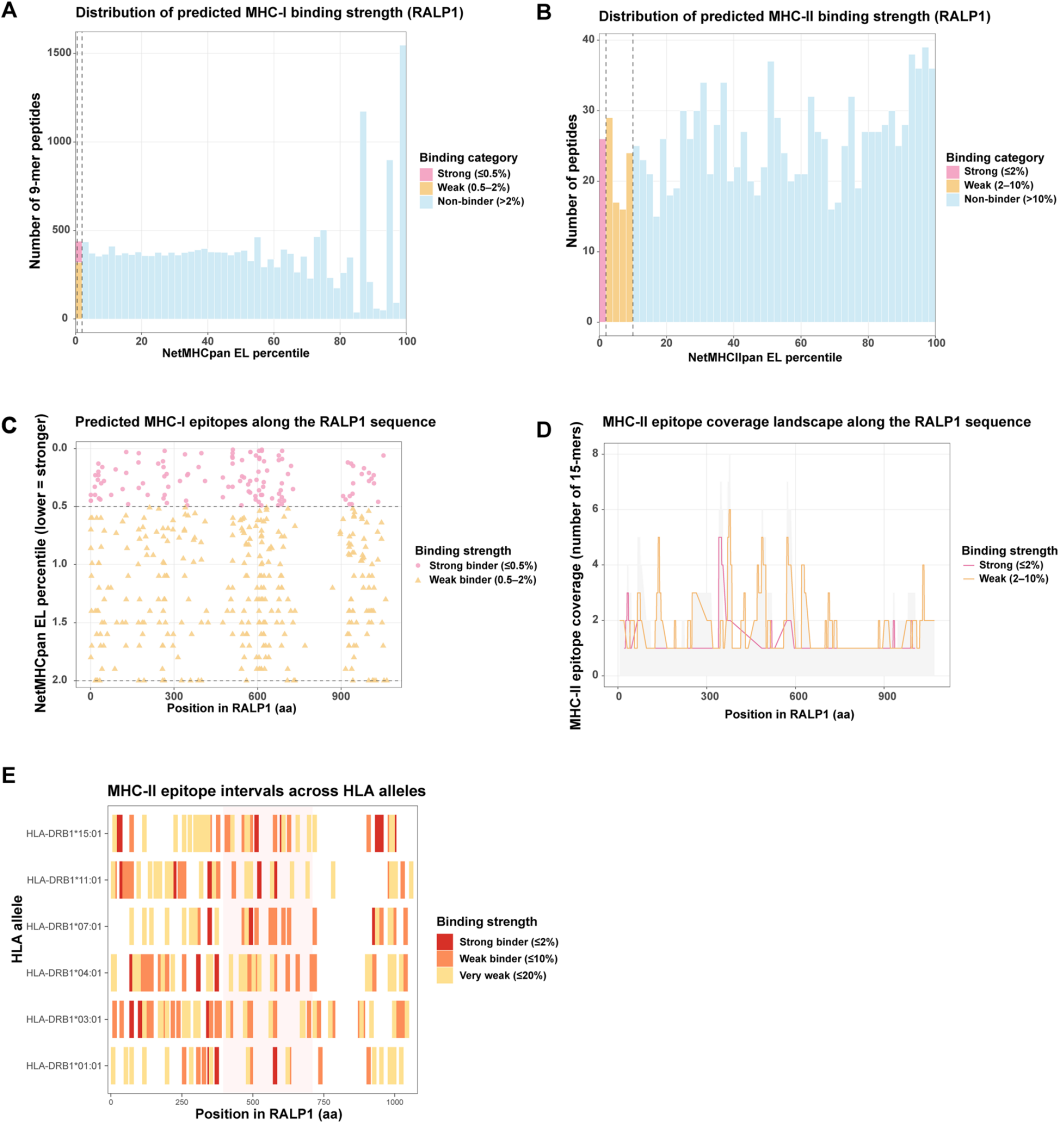
Predicted MHC-I and MHC-II epitope binding profiles across the RALP1 protein. **A** Distribution of predicted binding strength for all possible 9-mer MHC-I epitopes derived from the RALP1 sequence, categorized as strong binders (percentile rank ≤ 0.5%), weak binders (percentile rank 0.5–2%), and non-binders (percentile rank > 2%) based on NetMHCpan percentile ranks. **B** Distribution of predicted MHC-II binding strength for 15-mer peptides, classified as strong binders (percentile rank ≤ 2%), weak binders (percentile rank 2–10%), and non-binders (percentile rank > 10%). **C** Positional mapping of predicted MHC-I epitopes along the full-length RALP1 protein. Strong and weak binders are displayed according to their NetMHCpan percentile ranks (lower values indicate stronger predicted binding). **D** Coverage landscape of predicted MHC-II epitopes along RALP1, showing the number of 15-mer peptides overlapping each amino acid position for strong and weak binders. **E** Predicted MHC-II epitope intervals across representative HLA alleles. Strong, weak, and very weak binders are shown, illustrating the distribution of potential CD4⁺ T-cell epitopes along the RALP1 sequence.

To further evaluate the immunogenic potential of RALP1, we conducted a comprehensive in silico prediction of peptide binding affinity to human MHC class I and II molecules (Fig. 6). Using NetMHCpan and NetMHCIIpan algorithms, all possible RALP1-derived peptides were assessed and categorized into strong, weak, and non-binders. For MHC-I epitopes, affinity distribution analysis revealed a substantial number of candidate 9-mer peptides with intermediate to strong binding scores (Fig. 6A). Mapping the predicted epitopes along the RALP1 sequence demonstrated that strong MHC-I binders (percentile rank ≤ 0.5%) were broadly distributed, with a clear accumulation toward the C-terminal half of the protein, overlapping the experimentally reported RBC-binding domain (aa 396–710) (Fig. 6C). MHC-II binding predictions revealed a similar pattern. Although the total number of high-affinity 15-mer peptides was smaller than for MHC-I (Fig. 6B), strong and weak MHC-II binders formed discrete clusters within the C-terminal region (Fig. 6D). Epitope interval mapping across multiple HLA-DR alleles demonstrated that these C-terminal hotspots were consistently observed across diverse MHC-II backgrounds, including HLA-DRB1*15:01, HLA-DRB1*11:01, and HLA-DRB1*03:01 (Fig. 6E). Together, these analyses indicate that RALP1 harbors multiple T-cell epitope-dense regions—particularly within the C-terminal RBC-binding domain—suggesting that this region may contribute disproportionately to immune recognition and may warrant further investigation as a potential vaccine-relevant antigenic fragment.

## Discussion

RALP1 is a rhoptry neck-associated protein proposed to function during merozoite invasion, but its role has been difficult to define because *ralp1* is refractory to conventional gene knockout. Previous work showed that RALP1 localizes to the rhoptry neck in merozoites, dynamically relocalizes to the moving junction during erythrocyte invasion, and is not a core component of the canonical RON–AMA1 tight junction [16]. Antibody neutralization studies further suggested that targeting RALP1 can inhibit erythrocyte invasion and intraerythrocytic development [16]. However, the direct impact of RALP1 genetic manipulation on *Plasmodium* growth and invasion remained unclear. Importantly, whether RALP1 influences parasite development solely at the level of invasion or exerts broader effects on schizont maturation has not been systematically addressed.

In this study, we combined conditional genetics, phenotypic assays, transcriptomic profiling, and in silico analyses to dissect the function of RALP1 in *P. falciparum*. Using CRISPR-Cas9, we generated an inducible knockdown line by inserting a *ha-glmS* cassette at the 3′ end of *ralp1*. Upon GlcN treatment, Western blotting revealed a substantial reduction of RALP1 protein, confirming effective GlcN-inducible reduction of RALP1 expression. Under these conditions, parasite multiplication was markedly reduced over several asexual cycles, consistent with previous unsuccessful attempts to disrupt *ralp1* and with the essential roles reported for many rhoptry proteins during asexual RBC stages [14, 15, 31]. Notably, although *glmS*-mediated knockdown resulted in only a partial reduction (~ 40%) of RALP1 protein levels during the schizont stage, this decrease was sufficient to cause pronounced defects in schizont maturation and parasite proliferation, consistent with the dosage-sensitive roles reported for multiple rhoptry-associated proteins[32, 33]. Morphological analyses further showed that reduced RALP1 levels compromised schizont maturation and the reduced the number of merozoites per schizont. In synchronized invasion assays, ring formation was significantly decreased in RALP1-knockdown parasites, whereas egress from mature schizonts appeared less affected. Together with its dynamic relocalization from the rhoptry neck to the moving junction [16], these findings support a model in which RALP1 contributes to both proper merozoite formation and subsequent erythrocyte invasion.

To explore the broader consequences of partial knockdown of RALP1, we performed RNA-seq of synchronized *ralp1-ha-glmS* parasites with or without GlcN at early ring and late schizont stages. Most differentially expressed genes were detected at the schizont stage, consistent with peak RALP1 expression and the prominent schizont phenotype. This stage-specific transcriptional impact is unlikely to reflect a direct transcriptional regulatory role of RALP1 but instead suggests secondary consequences of impaired schizont maturation and defective assembly of invasion-related organelles. Taken together, the observed transcriptomic signature is most consistent with indirect consequences of impaired late schizogony and defective maturation of invasion-related apical organelles. Downregulation was the predominant pattern, suggesting a general attenuation of invasion and maturation programs rather than isolated changes. Notably, multiple members of invasion-associated gene families were reduced at the transcript level, including genes involved in erythrocyte interaction and apical organelle function. Although the specific identity of differentially expressed invasion-related genes was refined after correction for glucosamine-responsive transcripts, the overall pattern remained consistent, with broad suppression of gene families involved in erythrocyte interaction, membrane remodeling, and apical organelle function. These transcriptional changes provide a plausible molecular basis for impaired host cell binding and reduced fitness in parasites with reduced RALP1 expression.

Rhoptry proteomic studies have identified dozens of neck and bulb proteins in *P. falciparum* [14, 34]. Consistent with these annotations, our transcriptomic analysis showed that several rhoptry neck-associated genes—including members of the RH family (RH1, RH2a, RH2b, RH4, RH5), which function as major adhesins and include the essential PfRH5–basigin interaction [6, 35, 36]—were reduced in RALP1 knockdown parasites, with RH4 and RH5 displaying particularly pronounced decreases. Genes encoding components of the apical RON2/4/5 complex, which cooperates with AMA1 to form the tight junction [37], as well as additional neck-associated factors such as ASP, RON6, RON11, and RON12 [24, 38], were similarly downregulated.

Notably, multiple genes associated with rhoptry neck function and invasion-related apical machinery were consistently downregulated. Given that RALP1 itself localizes to the rhoptry neck, these changes are more plausibly interpreted as secondary consequences of impaired rhoptry neck assembly or maturation rather than evidence for a direct transcriptional regulatory role. Proper formation and coordinated secretion of rhoptry neck components during late schizogony require precise structural organization, and disruption of a neck-associated scaffold or organizer protein such as RALP1 is therefore likely to compromise this process, leading to downstream attenuation of invasion gene expression programs.

Beyond classical neck ligands, other apical and exported determinants—including SURFIN4.2, which traffics with PfEMP1 and RIFIN and localizes to an apical cap in free merozoites [39, 40], and ARO, an Apicomplexa-specific ARM-repeat protein required for proper rhoptry positioning [41, 42]—were also downregulated. In addition, several members of the REX (ring-exported protein) family, including rex1–4, were also downregulated following partial knockdown of RALP1. REX proteins are parasite-exported factors that localize to the host erythrocyte cytoskeleton and Maurer’s clefts, where they contribute to host cell remodeling and parasite virulence rather than direct receptor-mediated invasion [43]. The altered expression of multiple rex genes therefore further supports a broad perturbation of parasite programs involved in host-parasite interface remodeling downstream of impaired schizont maturation. These changes support a broader disruption of apical complex organization and virulence-factor export following partial knockdown of RALP1, contributing to defects in invasion and intraerythrocytic development.

We note that correction for nonspecific GlcN-induced transcriptional effects in this study was based on previously published RNA-seq data generated using 2.5 mM GlcN, whereas *glmS* activation in our experiments was performed with 5 mM GlcN. In this study, parallel RNA-seq analysis of wild-type parasites treated with 5 mM GlcN under identical experimental conditions was not performed. Because GlcN-associated transcriptional responses may be dose-dependent and cross-study comparisons are inherently subject to batch variation, direct equivalence between datasets cannot be assumed. Published studies evaluating multiple GlcN concentrations in wild-type *Plasmodium falciparum* have reported that 5 mM GlcN can lead to a modest delay in parasite growth but does not induce overt morphological abnormalities or severe developmental arrest under standard culture conditions [44]. Although these observations argue against severe nonspecific toxicity at 5 mM GlcN, we cannot formally exclude a contribution of dose-dependent GlcN effects to the observed transcriptional changes. Parallel RNA-seq analysis of wild-type parasites treated with 5 mM GlcN under identical experimental conditions would further strengthen discrimination between *glmS*-specific and GlcN-associated responses.

In addition to functional genetics and transcriptomics, we examined structural and antigenic properties of RALP1. AlphaFold3 prediction yielded an overall low pTM score (~ 0.23), indicating that RALP1 likely contains extensive intrinsically disordered regions. Such extensive intrinsic disorder is increasingly recognized as a common feature of invasion-associated proteins, enabling conformational flexibility and multivalent interactions during rapid host cell entry [45]. Together with its Gly/Glu-rich segments and leucine zipper–like domain, this supports a model in which RALP1 functions as a flexible molecular scaffold that can engage multiple partners at the rhoptry neck and moving junction. Here, “leucine zipper–like” is used strictly as a structural descriptor and does not imply a canonical DNA-binding function, consistent with our localization data.

From a structural perspective, our in silico analyses provide a framework for interpreting the functional behavior of RALP1 during erythrocyte invasion. The N-terminal and central regions are predicted to be largely intrinsically disordered, whereas the C-terminal region (approximately residues 396–710), previously implicated in erythrocyte binding, contains relatively more structured elements. This organization is consistent with a model in which intrinsically disordered regions confer conformational flexibility and support transient or multivalent interactions, while the C-terminal region may serve as a functional interface with host erythrocyte components. Epitope prediction highlighted several surface-exposed, disordered regions harboring putative B-cell epitopes, in agreement with previous reports that anti-RALP1 antibodies can neutralize invasion and inhibit intraerythrocytic development [16]. These features, together with its essential role during blood-stage development and prior reports of RALP1 antigenicity, suggest that RALP1 may represent a potential component for future multicomponent blood-stage vaccine studies, while acknowledging that direct experimental validation of protective efficacy remains required.

The *glmS* system produced partial rather than complete loss of RALP1, so the phenotypes observed likely reflect hypomorphic effects. The stage-dependent nature of the observed phenotypes is consistent with the mechanism and temporal dynamics of the *glmS* ribozyme system. Because *glmS*-mediated mRNA degradation results in gradual rather than acute protein loss, residual RALP1 is likely sufficient to support earlier intraerythrocytic stages, whereas defects become apparent during late schizont maturation and erythrocyte invasion, when functional demand is highest. The transcriptional alterations reported here may include both primary and secondary responses to partial knockdown of RALP1, underscoring the need for time-resolved or single-cell analyses to distinguish direct from indirect consequences. Furthermore, although our data indicate broad impacts on invasion ligands and apical factors, the direct interaction partners of RALP1 and its erythrocyte receptor(s) remain unknown. Future work should therefore focus on defining the RALP1 interactome using proximity labeling or co-immunoprecipitation, determining whether RALP1 contributes to rhoptry biogenesis or secretion and mapping the functional relevance of its C-terminal erythrocyte-binding domain. In addition, we note that the *ralp1-ha-glmS* parasite line used in this study was not cloned following genome editing. Although diagnostic PCR did not detect residual wild-type alleles at the population level, the presence of minor subpopulations with alternative integration events or low-level wild-type loci cannot be formally excluded. Such potential heterogeneity could, in principle, influence phenotypic or transcriptomic analyses. Validation using independently cloned parasite lines would therefore further strengthen the robustness of the observed findings.

## Conclusions

Our findings identify RALP1 as an essential rhoptry neck-associated factor that supports schizont maturation and erythrocyte invasion in *Plasmodium falciparum*. Partial knockdown of RALP1 disrupts key invasion programs and compromises parasite fitness, highlighting its importance for blood-stage development. Given its essentiality, localization, and antigenic potential, RALP1 represents a promising target for future antimalarial intervention strategies.

## Supplementary Information

Table S1. Primers were designed to amplify the homology arms for CRISPR/Cas9-mediated integration of the *ha-glmS* cassette at the 3′ end of the *ralp1* gene. Additional primers were used for diagnostic PCR to verify successful genomic integration in the *ralp1-ha-glmS* parasite.

Table S2. Differentially expressed genes (DEGs) in *Plasmodium falciparum* 3D7 parasites at the ring and schizont stages.

Table S3. Differentially expressed genes (DEGs) in RALP1-knockdown parasites at the ring and schizont stages

Fig. S1. ChIP-seq profiles of ApiAP2 transcription factors at the *ralp1* genomic locus.

Fig. S2. Morphological validation of parasite developmental stages at the time of RNA-seq sample collection.

Fig. S3. Identification of glucosamine-responsive genes and comparison with RALP1 knockdown–associated transcriptional changes. (A) Volcano plots of differentially expressed genes in synchronized parental *Plasmodium falciparum* 3D7 parasites cultured with or without glucosamine (+GlcN vs −GlcN) at the ring and schizont stages. (B) Venn diagrams showing the overlap between glucosamine-responsive genes identified in the parental 3D7 strain and differentially expressed genes detected in *ralp1-ha-glmS* parasites at the corresponding developmental stages, for both upregulated and downregulated gene sets.

Fig. S4. Predicted structural architecture of PfRALP1 by AlphaFold3.

## Data Availability

The RNA-seq dataset generated during this study has been deposited in GEO under accession number GSE298920. Reviewers can access the dataset using the private token qlgtmeomrdypnon.

## Abbreviations

AMA1: Apical membrane antigen 1
DBL: Duffy binding-like
EBA: Erythrocyte binding antigen
FPKM: Fragments per kilobase of transcript per million mapped reads
GlcN: Glucosamine
GO: Gene ontology
HLA: Human leukocyte antigen
IDRs: Intrinsically disordered regions
MHC: Major histocompatibility complex
MoRFs: Molecular recognition features
PVM: Parasitophorous vacuole membrane
PV: Parasitophorous vacuole
RALP1: Rhoptry-associated leucine zipper-like protein 1
RAMA: Rhoptry-associated membrane antigen
RAP: Rhoptry-associated protein
RBC: Red blood cell
RBL: Reticulocyte binding-like
RESA: Ring-infected erythrocyte surface antigen
RH: Reticulocyte-binding-like homologue
RNA-seq: RNA sequencing
RON: Rhoptry neck
Rhop: Rhoptry
SURFIN: Surface-associated interspersed protein
TJ: Tight junction
