# Supplementary figures and images for: RALP1 is essential for schizont maturation and erythrocyte invasion in Plasmodium falciparum
Source: Parasit Vectors. 2026 Mar 6;19:158. doi: 10.1186/s13071-026-07329-w (PMC13078051; doi:10.1186/s13071-026-07329-w)

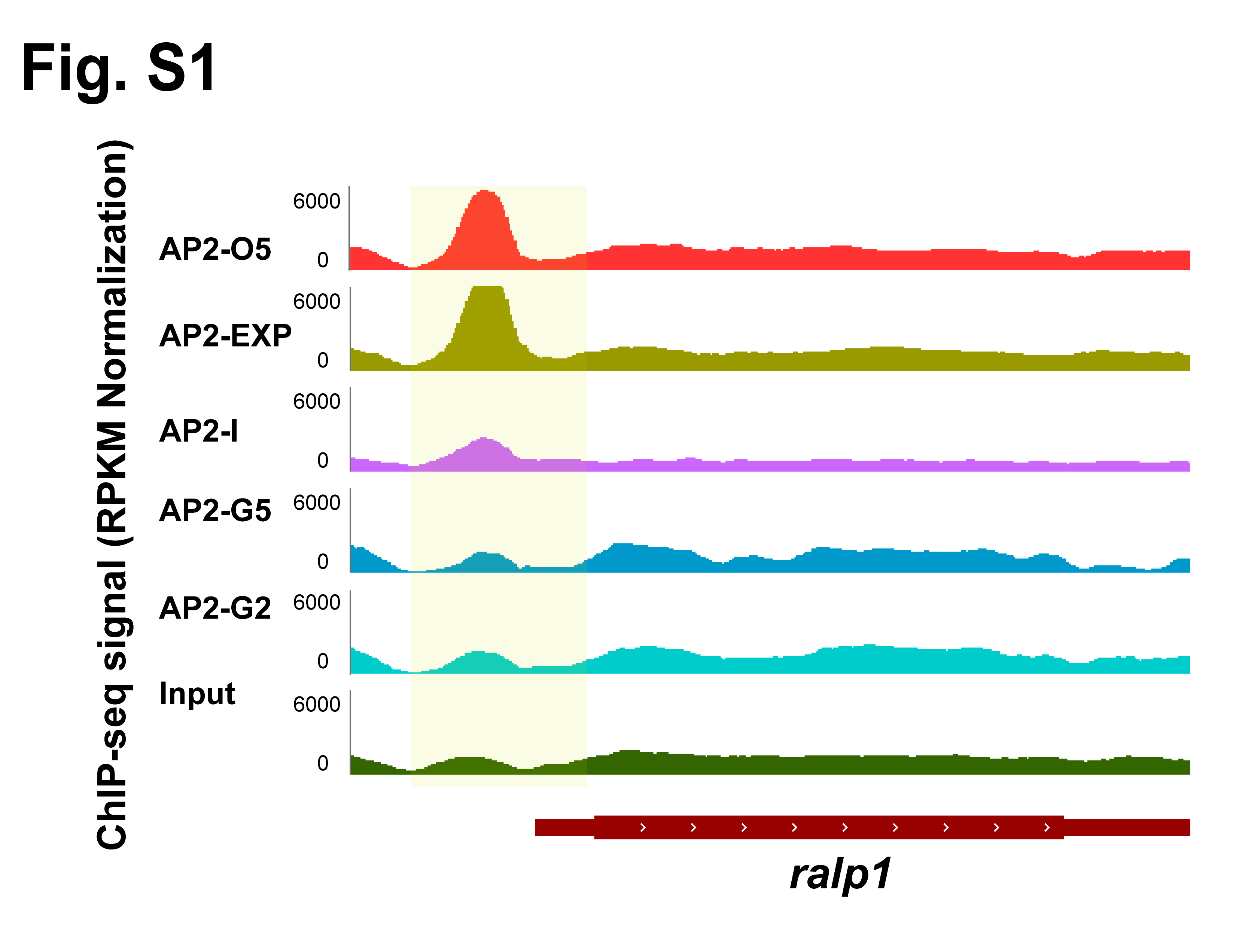

Supplement: Supplementary file 4 — Fig. S1. ChIP-seq profiles of ApiAP2 transcription factors at the ralp1 genomic locus. [file 13071_2026_7329_MOESM4_ESM.tif]

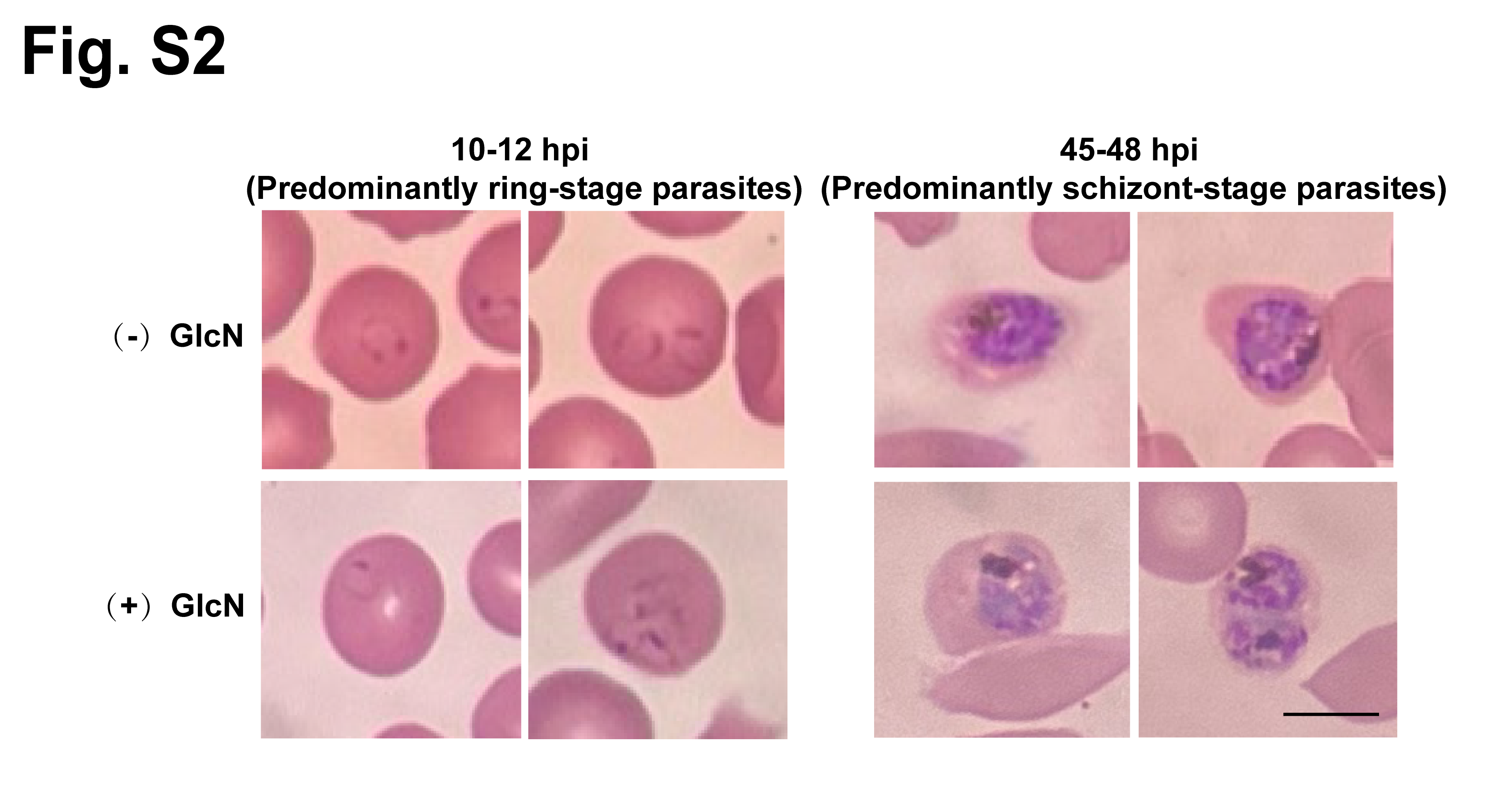

Supplement: Supplementary file 5 — Fig. S2. Morphological validation of parasite developmental stages at the time of RNA-seq sample collection. [file 13071_2026_7329_MOESM5_ESM.tif]

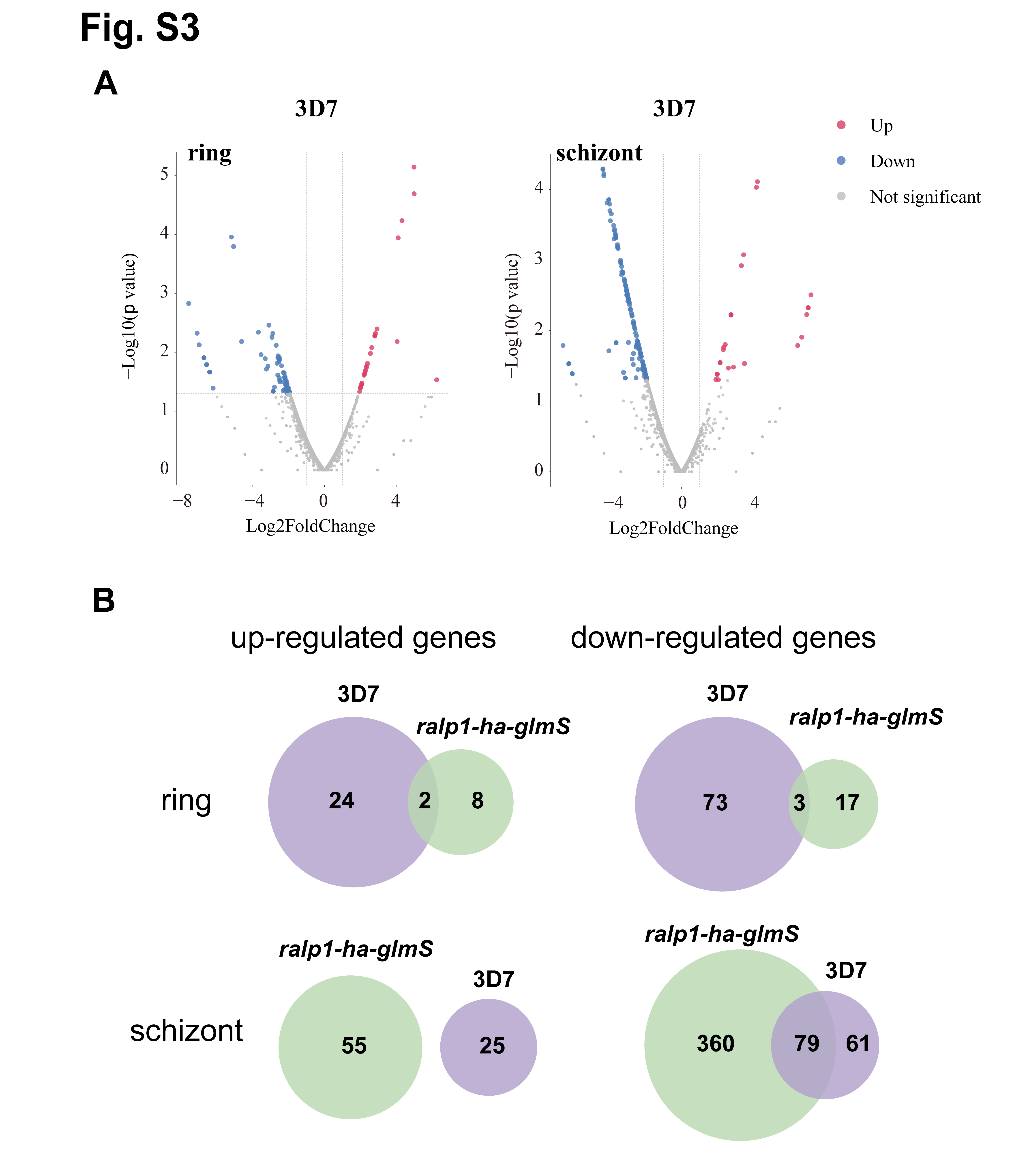

Supplement: Supplementary file 6 — Fig. S3. Identification of glucosamine-responsive genes and comparison with RALP1 knockdown–associated transcriptional changes. (A) Volcano plots of differentially expressed genes in synchronized parental Plasmodium falciparum 3D7 parasites cultured with or without glucosamine (+GlcN vs −GlcN) at the ring and schizont stages. (B) Venn diagrams showing the overlap between glucosamine-responsive genes identified in the parental 3D7 strain and differentially expressed genes detected in ralp1-ha-glmS parasites at the corresponding developmental stages, for both upregulated and downregulated gene sets. [file 13071_2026_7329_MOESM6_ESM.tif]

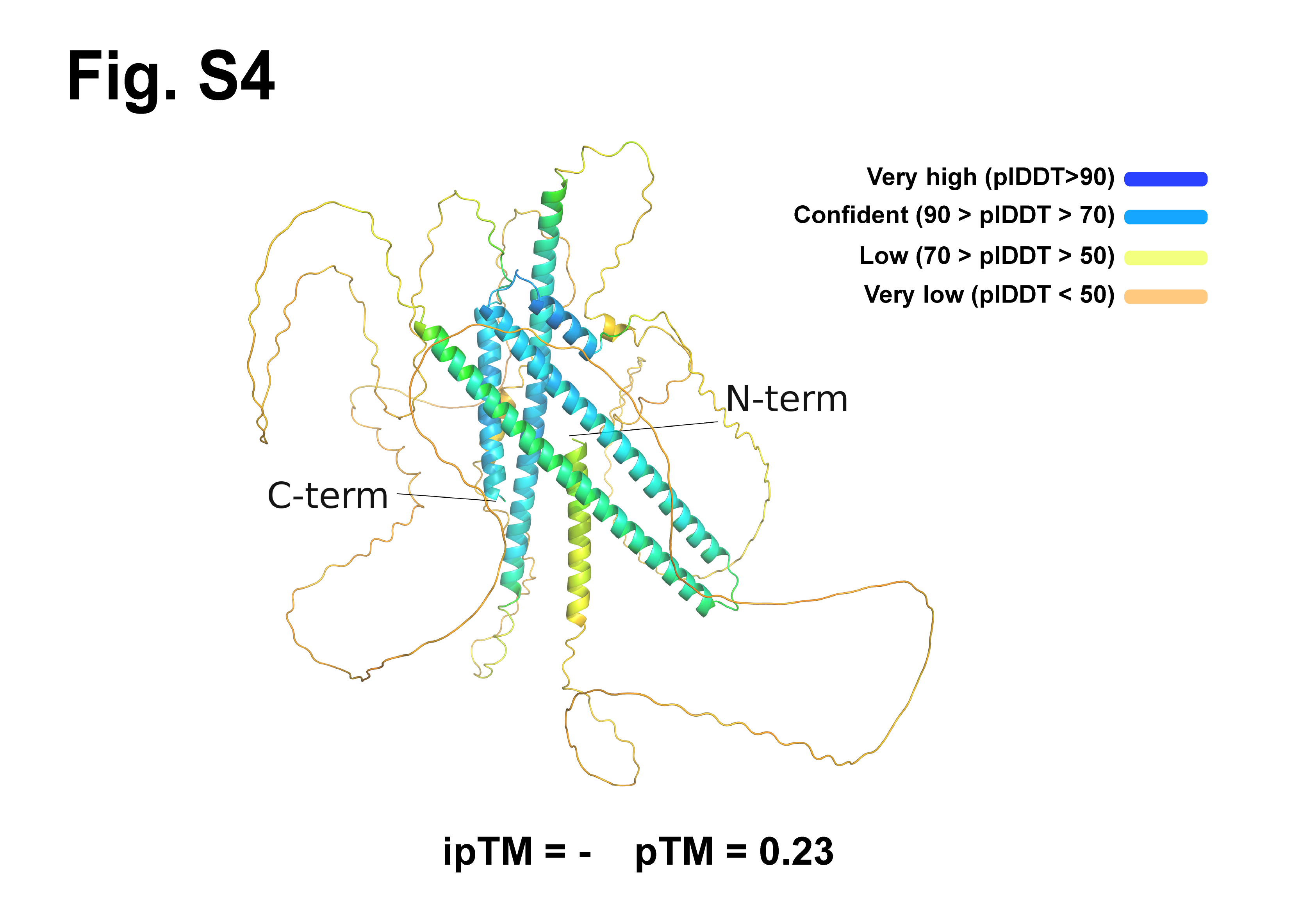

Supplement: Supplementary file 7 — Fig. S4. Predicted structural architecture of PfRALP1 by AlphaFold3. [file 13071_2026_7329_MOESM7_ESM.tif]
